# What does it take for healthy food retail programmes to be successful? Lessons learned in New York City

**DOI:** 10.1017/S1368980024001368

**Published:** 2024-10-03

**Authors:** Felicia J Setiono, Samantha P Heller, Tashara M Leak

**Affiliations:** Division of Nutritional Sciences, Cornell University, Ithaca, NY, USA

**Keywords:** Food environment, Urban, Food stores, Healthy retail programmes, Corner stores

## Abstract

**Objective:**

Healthy food retail programmes (HFRP) in the USA generally aim to increase healthy foods access to improve diet quality and health, yet the impact is mixed. These programmes primarily target adults, even though adolescents frequently and independently visit stores to purchase snacks. This study’s aims are to explore successes and challenges of implementing HFRP (Aim 1) and examine how HFRP can be tailored to adolescents (Aim 2).

**Design:**

One-time, virtual, semi-structured interviews with individuals who were involved in a HFRP, followed by a socio-demographic characteristics survey. Interviews were designed based on the RE-AIM framework and the Hexagon Tool and analysed using Braun and Clark’s (2006) thematic analysis approach. Descriptive statistics were used to summarise participants’ socio-demographic characteristics.

**Setting:**

New York City (NYC).

**Participants:**

Adults (18 years or older) who have designed, implemented and/or evaluated an HFRP in NYC and speak/understand English (*n* 21).

**Results:**

Aim 1: For successes, strategies to build relationships with the community were most discussed. Regarding challenges, securing reliable funding was the hardest to overcome. Suggested solutions included designing profitable HFRP, targeting shortcomings in food distribution systems and increasing consumer demand. Aim 2: Most participants had not considered adolescents in previous HFRP but suggested involving youth in developing HFRP to encourage youth-driven solutions and promote youth advocacy.

**Conclusions:**

Future HFRP should focus on activities that help store owners purchase affordable healthy foods from distributors, which translates to affordability for customers. Federal and local policies can assist by funding complementary programmes. Additionally, adolescents should be considered in these efforts.

Public health practitioners and nutritionists are increasingly working to create food environments that promote the purchase and consumption of healthy foods, such as fruits and vegetables^(1,2)^. Research has generally shown that an individual’s access to healthy foods in their neighbourhood is positively associated with their dietary consumption^(3,4)^. For example, higher access to supermarkets has been shown to be associated with fruits and vegetables consumption, whereas higher access to corner stores is associated with intake of energy-dense nutrient-poor items such as sugar-sweetened beverages (SSB)^(2,5,6)^. Experts recognise however that other factors, such as habits, preference and transportation access, may influence individuals’ food purchasing decisions^(7,8)^. Still, improving the food environment is considered a promising avenue to address poor diet quality, which is known to be a leading risk factor for chronic diseases and mortality^(9–11)^.

In the USA, disparities in the food environment are said to contribute to disparities in diet quality and health^(12)^. Supermarkets are more often found in neighbourhoods predominantly inhabited by high-income individuals who identify as White, compared to in neighbourhoods whose residents are majority low-income and identify as a racial/ethnic minority^(2,13)^. The reverse is true for the density of corner stores and fast food places^(13,14)^. Additionally, a recent study revealed that between 1990 and 2014, the amount of traditionally unhealthy food sources (e.g. corner stores) largely increased in neighbourhood tracts that were not predominantly non-Hispanic White^(15)^. This disparity in the food environment parallels the disparities in chronic diseases observed between racial/ethnic groups^(16,17)^.

In light of this knowledge, healthy food retail programmes (HFRP) have been suggested as one way to reduce disparities and improve the food environment. Most HFRP have been implemented in urban areas^(18)^. This is unsurprising as compared to those in rural areas, people who live in urban areas have higher access to supermarkets/grocery stores, as well as less healthy retailers^(14,19,20)^. The activities of an HFRP can differ greatly, but the goal is usually to increase the availability and accessibility of healthy foods in food retailers, particularly in corner stores^(21)^. For example, one study conducted in Baltimore, Maryland, worked with corner stores to display caloric information on labels^(22)^. Another programme in New York City (NYC) worked with corner stores to make various changes, which included displaying water at eye level and stocking foods such as dark green leafy vegetables^(23)^. Improvements in product placement, labelling and advertising are similarly typical of HFRP that occur in supermarkets^(24–27)^. For example, in the UK, several programmes worked with supermarkets to remove display units for confectionaries from prominent areas and instead increase the visibility of fruits and vegetables^(26,27)^.

What is currently lacking is knowledge of what factors facilitate HFRP success. Research has shown that some programmes improved purchasing behaviour, while others had no impact^(21,25,28)^. Elucidating the reasons for the mixed results is limited as most HFRP studies do not report on the factors that contribute to programmes’ successes and/or challenges. Additionally, the variety in the design and activities of existing HFRP makes comparisons across programmes difficult^(25)^. Efforts to understand and increase the success rates of HFRP are important since corner stores play an important role in providing food for urban residents. In a survey of adult shoppers in NYC, 68·5 % reported visiting the corner store at least daily^(29)^. Another study with over 2000 adults in NYC found that 35 % of respondents bought most, if not all of the food they consumed in a month at corner stores^(30)^.

To note, most HFRP have focussed on adult customers^(21,25,28)^, but adolescents are also an important customer base for corner stores. Many urban corner stores are located near middle and high schools, creating easy access for adolescents to visit^(31)^. A study with more than 2000 adolescents in the USA found that almost half visit corner stores at least weekly^(32)^. In urban cities, visits to corner stores are more frequent^(33,34)^. For example, a study in Baltimore found that adolescents were visiting corner stores on average twice a week, with energy-dense nutrient-poor items being the most commonly purchased items^(33)^. Despite this, it remains unclear how to most effectively tailor HFRP for the adolescent population.

To address the gaps above, we conducted qualitative interviews with researchers and practitioners involved in HFRP in NYC, the largest urban city in the USA^(35)^. We sought to answer the following research questions: (1) what factors contribute to the successes and challenges of implementing HFRPs in NYC? and (2) how can healthy retail programmes be tailored to encourage healthier food purchasing among adolescents?

## Methods

### Setting

Corner stores play a large role in providing foods for NYC residents, carrying close to 20 % of the food volume available in the city^(36)^. Across city neighbourhoods, the ratio of corner stores to supermarkets varies highly, a variation that overlaps with differences in the neighbourhood racial makeup. In Bedford-Stuyvesant, where more than 75 % of residents are non-White, there are fifty-seven corner stores for each supermarket present^(37)^. On the Upper West Side, where 66 % of residents are White, the ratio drops to three corner stores for each supermarket^(37)^. As a result, there has been an abundance of HFRP focusing on either corner stores and/or supermarkets in NYC. Some examples of HFRP in NYC, which may or may not be eventually represented in this study’s sample are listed in Table 1.

**Table 1:** Examples of healthy food retail programmes (HFRP) based in New York City (NYC) that were active sometime between the years 2007 and 2023*

HFRP name	Sponsor	Programme overview
Healthy Bodegas Initiative^(38)^	NYC Department of Health and Mental Hygiene	Target primarily corner stores in low-income neighbourhoods with a high prevalence of chronic illness Increase stocking of nutritious foods e.g. fresh produce, whole grain products, low-fat and fat-free milk Labelling and placing nutritious items in visible and prominent areas in stores
Shop Healthy† ^(39)^	NYC Department of Health and Mental Hygiene	Target a variety of food retailers, suppliers/distributors and community organisations in low-income neighbourhoods with a high prevalence of chronic illness Work with food retailers to set goals to stock, place and promote nutritious foods in stores Ask suppliers/distributors to promote healthier foods
Get the Good Stuff^(40,41)^	NYC Department of Health and Mental Hygiene	Work with supermarkets to offer an incentive programme for Supplemental Nutrition Assistance Program (SNAP) recipients to purchase fruits, vegetables and legumes SNAP recipients who buy eligible food items earn up to $10 a day back in Electronic Benefit Transfer that can be used for their next qualified purchase
Food Retail Expansion to Support Health^(42)^	NYC Economic Development Corporation	Target supermarket operators and developers in areas lacking access to healthy foods Provide tax breaks to build or renovate supermarkets in eligible areas
Healthy Retail Programmes^(43–45)^	City Harvest	Focus on supermarkets and corner stores Offer food retailers updated signage and displays to promote healthy foods Provide training on storage of fresh produce
Supermarket Tours^(46)^	City Harvest	Target customers at supermarkets Provide “Cooking Matters at the Store” tours to educate shoppers on how to read food labels, find healthy products and budget their grocery list Offer a $10 incentive for customers to choose groceries in line with MyPlate
SNAP-Education (SNAP-Ed)^(47,48)^	BronxWorks	Work with a variety of food retailers (e.g. corner stores, supermarkets, vendors) in the Bronx Host cooking demonstrations on how to prepare healthy meals and nutrition lessons at food retailers

* HFRP listed in this table may or may not be represented in the current study.

† Shop Healthy was built upon lessons learned from the Healthy Bodegas programme and became its next iteration. Hence, they overlap in purpose and activities.

### Participants and recruitment

Individuals who have been involved in the development, implementation and/or evaluation of an HFRP in NYC were recruited to participate in a one-time semi-structured interview. For the purpose of this study, recruitment of researchers and practitioners who worked on HFRP at corner stores and supermarkets was prioritised, as most HFRP have targeted these food retailers. Programmes could have been funded privately (e.g. through a research grant) or publicly (e.g. federal/state grants). Participants had to be able to speak/understand English and be 18 years or over to participate. If multiple people are working or have worked on the same programme, the first five people within the same programme who completed the consent form were enrolled in the study. Participants received a $50 incentive in the form of an electronic gift card.

Participants were recruited in three main ways: (1) a literature review of journal articles in the past 15 years (since 2007) that have reported on the design, implementation or evaluation of HFRPs conducted in NYC. The following keywords ‘(program* OR interven*) AND (retail* OR food* OR beverage* OR drink* OR soda* OR snack*) AND (store* OR shop*) AND (New York* OR NYC)’ were entered into several databases including PubMed, Web of Science, EconLit and Pyschinfo. Two researchers conducted the review and identified potentially relevant articles independently. The researchers met to remove duplicates and articles that lacked relevance. The first and last authors of each selected article were contacted as potential participants. In cases where the articles were written in collaboration with a non-academic organisation, the first listed author who belongs to the organisation was also contacted. (2) Researchers and organisations in retail work who previously collaborated with this study’s last author were contacted. (3) Snowball sampling technique was also used in that participants were asked to recommend other people they know who might be suitable participants^(49)^. Contacted individuals received an email with the study’s information and recruitment flyer. Upon expressing interest, they were screened for eligibility.

### Data collection

Interviews were conducted virtually from September 2022 to January 2023 using either Zoom or phone, depending on participants’ preference. All interviews were conducted in English. At the end of the interview, respondents completed a short survey on socio-demographic characteristics (age, sex, racial identity, ethnicity) as well as their roles/position, amount of time spent on the programme and affiliations when they worked for the HFRP. Role/position was asked as a free-response question (‘What was your role/position when you worked on the retail programme?’). Affiliation was asked as a multiple-choice question with options of ‘University/college’, ‘Government’, and ‘Non-profit organization’ and ‘Other’.

### Interview questions

A semi-structured interview guide was developed using the Reach, Effectiveness, Adoption, Implementation and Maintenance (RE-AIM) Framework^(50)^, commonly used to evaluate health programmes^(51)^. Another framework called the Hexagon Tool^(52)^ was used to supplement the interview guide to develop questions specific to resources and logistical considerations needed in planning programmes. The Hexagon Tool^(52)^ assesses six constructs: Need, Evidence, Fit, Usability, Capacity and Support. Definitions of the frameworks’ constructs are described in Table 2. along with corresponding interview questions. In addition, participants were also asked, ‘If we want to tailor the programme to target adolescents’ purchasing behaviours, what additional recommendations would you have?’.

**Table 2:** Definition of the RE-AIM framework and the hexagon tool constructs as they relate to healthy food retail programme evaluation with corresponding interview questions and probes used in the study*

	Framework	Constructs	Definition/scope	Interview questions/probes
	RE-AIM	Reach	The extent that the programme reached the intended population (stores and customer base)	What criteria did you have for stores to participate in the (healthy food retail programme)?
		Effectiveness	Impact/outcomes of the healthy food retail programme	What were some changes or impact you wanted to see from the programme? Going back to the programme’s original goals/impact, what were some of the changes you observed for stores participating in the (healthy food retail programme)? What other measures would you have wanted to collect data on?
		Adoption	Willingness of stores reached to initiate the programme	What are some things that motivated stores to participate? What are some things that you could’ve done better to encourage participation? What are some barriers to participation? e.g. timing/seasons, lack of advertising, lack of incentive
		Implementation	Fidelity and consistency of the healthy food retail programme delivery	During the programme, what challenges did the (healthy food retail programme)/stores face? Can you tell me about any unexpected events that happened? How would you address these things if they were to happen again?
		Maintenance	The extent of healthy food retail programme being sustained long-term	What is the status of the (healthy food retail programme) now? What actions may help maintain the programme? What are some barriers to maintaining the programme? In an ideal world, how would you go about ensuring that the programme can have lasting impact? Or be sustained longer?
	Hexagon Tool	Need	Identifying community needs and population/ subpopulation to focus on	Can you talk about how stores were involved in the development of the programme? What criteria did you have for stores to participate in the (healthy food retail programme)?
		Evidence	Evaluating the impact, fidelity and cost-effectiveness of the healthy food retail programme	What were some changes or impact you wanted to see from the programme? Going back to the programme’s original goals/impact, what were some of the changes you observed for stores participating in the (healthy food retail programme)? What other measures would you have wanted to collect data on?
		Fit	Aligning the healthy food retail programme components to fit community values and context	How did partners/stakeholders contribute to the development of the (healthy food retail programme)? (tell me more about who they are)
		Usability	Defining the healthy food retail programme activities clearly	Can you describe to me how the (healthy food retail programme) was created? Can you walk me through the typical activities or events of the programme?
		Capacity	Identifying the costs and resources needed for implementation of the healthy food retail programme	How was the programme supported financially? What are some actions that led you to secure enough funding for the programme? Based on this experience, what financial decisions or financial priorities would you make for future programmes?
		Support	Listing additional support needed to carry out the healthy food retail programme, e.g. training for staff	What kind of training was offered (for team staff and store staff)? What types of training would you keep and what other training do you wish to see if the programme was repeated? Knowing what you know now, are there things you would have done differently regarding training? Is there anything else that I should know about activities that were done in preparation of the (healthy food retail programme)?

RE-AIM, Reach, Effectiveness, Adoption, Implementation and Maintenance.

* Interview questions and probes may be listed more than once as they fit multiple constructs.

### Analysis

Interviews were audio-recorded and transcribed through Zoom auto-transcription or professionally by 3Play Media if conducted via phone. Transcripts were checked for accuracy and analysed using the thematic analysis approach outlined by Braun and Clarke (2006)^(53)^, which allows researchers to use theoretical frameworks to guide analysis. The coding process was as follows^(53)^: (1) two coders (author1, author2) went through transcripts independently to familiarise themselves with the data and created a memo of initial code ideas. (2) Coders independently open-coded transcripts and created memos on ideas they deemed important and/or salient. (3) Coders met to discuss their highlighted codes/ideas, identified themes and sub-themes based on the open codes and developed a coding scheme using a codebook (a table of codes, their definition and examples). (4) Coders coded all remaining transcripts using the new coding scheme and created memos on the coding process. Coders met weekly to discuss new ideas, refine themes and edit the codebook iteratively. (5) Coders met to map out how themes relate to one another and extract relevant quotes for each theme. (6) Tables were then used to summarise results. Coding was done in NVivo 12 (QSR International, USA). Descriptive statistics on socio-demographic characteristics from the post-interview survey were calculated using STATA 14.

### Trustworthiness and rigor

Interview guide was pre-tested with three participants with expertise in the research area. Based on the pre-test interviews, the guide was amended to clarify questions’ meaning, building credibility^(54)^. Two researchers coded interviews independently and met together to reflect on the process of coding, which helps maintain confirmability of results^(54)^. Additionally, the researchers wrote memos to reflect on their coding process as well as potential biases during analysis. This helps to build overall trustworthiness by developing a detailed and transparent record of how the researchers approached the interviews^(54)^.

## Results

Out of the thirty-nine individuals directly invited to participate in the interview, twenty-one completed the interview (54 % response rate). Participants represented eleven different HFRP in NYC that primarily work with corner stores, supermarkets or both types of stores. The socio-demographic characteristics of participants are presented in Table 3. On average, interviews lasted 45·8 (sd = 11·7) minutes.

**Table 3:** Socio-demographic characteristics of participants interviewed (*n* 21)

Socio-demographic characteristics	Frequency (%) unless otherwise stated	
	Mean or *n*	sd or %
Age (Mean (sd))	49·8	13·0
Gender		
Male	6	28·6 %
Female	15	71·4 %
Ethnicity		
Hispanic, Latino or of Spanish origin	6	28·6 %
Not Hispanic, Latino or of Spanish origin	15	71·4 %
Race		
Asian	1	4·8 %
Black or African American	3	14·3 %
White	13	61·9 %
Other	4	19·0 %
Affiliation		
College/University	7	33·3 %
Government	7	33·3 %
Non-profit organisations	7	33·3 %
Phase of involvement in HFRP		
Development (e.g. brainstorming ideas, securing funding)	16	76·2 %
Implementation (e.g. organising team, labelling foods at stores)	19	90·5 %
Evaluation (e.g. collecting data, interviewing)	12	57·1 %

HFRP, healthy food retail programmes.

Several themes were identified across the two research questions and were explored more in-depth in subsequent sections. In terms of understanding what factors contribute to the successes and challenges of implementing HFRP in NYC, participants discussed (1) strategies to build relationships to produce effective HFRP, (2) challenges in acquiring consistent levels of funding, (3) modifying HFRP to be a profitable venture and (4) considering systems-level factors that dictate the success of a HFRP. As for how HFRP can be tailored for adolescents, participants emphasised including youth in the process of developing HFRP to better target relevant food and retailer types.

### Suggested strategies may not be cost-effective

Participants stressed the importance of relationship building and how having a good relationship with store owners contributes significantly to the fidelity of the programme. As one interviewee explained

*“…a lot of our success had to do with the relationship that our staff built with the stores….our staff was going to the stores regularly. They became friends with the store owners, they built a trusting relationship [that] allowed us to do so much that I think we would not have been able to, had we not invested that time.” (Participant #14, Programme #4)*

Having staff who come from the community where the stores are located facilitates trust and encourages store owners to participate in HFRP. When staff share the language and culture of store owners and/or resemble the store’s customer base, store owners are usually more at ease. The following quotes demonstrate the contrasting attitude store owners can have when first recruited to be part of the programme:

*“If I walk in there and I know our culture is similar, I would pick on some of the foods and go, oh, you carry this? oh my God, this was my favorite growing up. Oh my God, where are you from? Just go to the person and get to know who they are… they’ll say oh she actually cares, she wants to know where I’m from. And most of the time people are happy to tell you where they’re from.” (Participant #9, Programme #10)*

*“I’ve walked into a lot of bodegas, and you know people don’t want to talk to you. People claim they don’t speak English. It’s just you know, ‘who are you?’. You just walked in off the street right. You don’t belong here, and you’re asking a lot of questions.” (Participant #15, Programme #3)*

The other important aspect of relationship building is frequent follow-ups and demonstrating one’s commitment to the stores. This should involve interacting with store owners in matters outside the scope of the HFRP. As one interviewee explained

*“If it’s Father’s Day, or holiday, you let them know. Hey, we’re thinking of you, or send them cards during the holiday. It’s important, if you’re not just looking at them as like…where can I use them, or how can I ask them to sign up for it? It’s also okay to reach out to them, even if you’re not asking them to do something for you. You don’t want to just be there for them whenever you need them.” (Participant #16, Programme #4)*

While most participants agreed that the time invested in relationship building was important, many were concerned about the cost associated with such activities. Relationship building required a lot of time and a diverse set of staff. Many programmes ended up with limited reach because of the amount of time needed to recruit a single store. When asked about challenges, one interviewee said

*“The cost, as a community intervention, the cost of the labor time and the staff time to win over bodega owners and get them to change was not insignificant….And that they ended up, having to live with a smaller number of stores that were actively engaged in the program to maintain the level of intensity of the intervention.” (Participant #2, Programme #10)*

### Funding flaws promote funding insecurity

Most participants mentioned that funding available for HFRP is usually limited in scope and duration, which has required them to find creative ways of partnering with other organisations to supplement programme activities. One interviewee stated

*“There was one big hospital in the area that also had some funding to work with corner stores, so we partnered with them and gave them shopping bags…and we also had a grant from another partnership, where they provided a refrigerator to a store…And this is why partnership is so important. Because they tell us, ‘oh, you have to go do all these changes’, but then there’s no money right or funding for it, so that’s when the creative part comes.” (Participant #4, Programme #8)*

It is common that multiple organisations in one community receive funding to implement similar programmes, which results in duplication of efforts that may be redundant rather than complementary. All of this can cause confusion for participating stores to understand what being a healthy food retailer means. One interviewee explained

*“I don’t think it’s particularly helpful for [organization 1] to go into Bodegas in ten blocks in the Bronx and for [organization2] to go into a different ten blocks and have different signage, and maybe have some of the same things and some not. There’s no coordination. I mean, there’s some coordination, but there’s also ‘I’m going to do it my way, because this is how I get my grant’.” (Participant #17, Programme #10)*

Another obstacle to securing funding is the restrictive definition of what it means for a programme to be successful. Funders can require reporting of certain metrics and failure to meet those expectations often comes with a penalty or discontinuation. The issue is some measures may or may not be attainable or even appropriate to capture a programme’s activities. One interviewee shared an instance where

*“[a funder] wanted us to track BMI And you’re like. No, we’re not gonna change BMI. I think we didn’t get the money again. But really what we were trying to impact was access. And so, we did most of our evaluations around that…at the beginning, people felt like they couldn’t find healthy food. At the end, they felt that they could. Did they buy it? Did they get healthier? That’s not what we’re doing, and that’s hard. Because if you’re not doing that, then, people might feel like it’s a waste.” (Participant #10, Programme #6)*

### Making healthy food retail programmes profitable: addressing stores’ concerns and promoting sustainability

Almost every participant found that HFRP are more attractive and adoptable if they are presented as a way to further store profit. While store owners are usually very committed and motivated to providing healthy foods to their community, they also fear the loss of profit and product waste.

*“We tried to use the community support angle. But really business case does the most…saying that you’re creating a necessary resource for a community was big…But it’s definitely secondary to the business case.” (Participant #13, Programme #9)*

Several suggestions on making HFRP more profitable and self-sustaining were made. One is to encourage the use of value-added products such as healthy smoothies or sandwiches. Value-added products have a higher profit margin and can sometimes be a way to use products that otherwise would be thrown away. Another is choosing activities that can promote healthy food stocking beyond the programme’s timeline. One example is providing the necessary infrastructure that stores need to stock perishable products (e.g. refrigerators and/or storage units). Others have also conducted training for store staff in techniques on how to procure different types of foods, keep fruits and vegetables fresh and how to make healthy value-added products. Examples include

*“Anything that can be like infrastructure or things that just last without you checking on them every two seconds are the best…recognizing the sustainability and how important that is, and that that will come with money and capital, and less about trying to convince people.” (Participant #10, Programme #6)*

*“We provide a lot of training, how to maintain the produce, but also resources and materials to keep it nice and presentable…checking your inventory, checking your produce, expanding your produce.” (Participant #16, Programme #4)*

### Rethinking what a healthy food retail programme looks like: invest in systems beyond the stores

There were differences in how participants approached the question of recommendations for future programmes. Participants who were in a supervisory role (e.g. director, programme managers) often advocated for reframing and rethinking of what HFRP looks like. Participants discussed the importance of addressing factors that prohibit the selling of healthy foods in stores at the distribution, stores and customer levels. These barriers must be addressed before repeating HFRP in their current model.

One major barrier is that the current food retail supply chain limits the ability of smaller stores to stock healthy foods at an appropriate price. Corner stores often lack an economy of scale; since they have limited space, they rarely buy food in bulk. Distributors are thus less amenable to providing food for corner stores compared to larger stores such as supermarkets. If this system is not addressed, it will be difficult to sustain HFRP as either the stores or consumers will end up bearing these added costs. An interviewee who worked with distributors said

*“They [distributors] do not have a lot of fun sourcing produce for smaller stores… they don’t want to break it down into the smaller batches to sell to the stores. They said it’s more expensive.” (Participant #11, Programme #6)*

Furthermore, smaller stores have limited staffing and usually lack capacity to travel and procure foods from distributors. Thus, suggestions were made to set up new distribution systems where corner stores can combine their purchasing power to purchase healthy foods in bulk. Delivery of these foods can potentially be made to a centralised location to be broken down, before being distributed to single stores to reduce the burden of store staff having to commute to procure products themselves. An interviewee described a similar system that exists in NYC’s Chinatown

*“in Chinatown…there were guys who ran trucks from either from the countryside or from Hunts Point [a distribution hub], got the fruits and vegetables, and delivered them at pretty low price points to retailers in Chinatown. And that kind of distribution network existed and was really serving those retailers.” (Participant #2, Programme #10)*

Another important consideration is the process by which stocked foods make it to corner store shelves. At present, food and beverage companies that distribute products to corner stores often hire contracted individuals to position and merchandise their products in each store. Merchandising products are common in existing HFRP, but such activities are time-consuming and burdensome for store owners/staff. Thus, a similar market could be developed where the responsibility of merchandising healthy foods is placed upon an external individual working as a mediator between distributors and stores. As one interviewee described

*“the salespeople really act as sort of like an additional employee. They come in, they merchandise, they keep track of what’s being sold. They restock because they work on commission.” (Participant #5, Programme #7)*

As mentioned, investments in infrastructure that enable long-term stocking and storage of healthy foods are important. Other capital funds may be needed to clean stores (e.g. removing advertisements of competitive foods from windows) and ensure that equipment can continue to be used (e.g. making sure stores can pay their electric bill). An interviewee who worked with a supermarket shared their experience

*“you can reduce the electricity bill for the supermarket operator, so they’re not passing down as much of that cost to their customer. I had talked to a supermarket operator who had this really really huge energy, inefficient refrigerators from the seventies. He replaced them and he was able to double the size of his produce section.” (Participant #13, Programme #9)*

Many participants mentioned price/affordability to be the number one driver for consumers’ decisions. Thus, suggestions to increase demand include advocating for policies to discount healthy products and disincentivise purchases of competitive foods through taxes. These two approaches can even be combined, as one interviewee suggested

*“if you designed a soda tax for a jurisdiction…If you funnel it back into a SNAP (Supplemental Nutrition Assistance Program)-like incentive program, the money goes back into people’s pockets, they’re going to spend at your store still, and then neutralize frankly some of the push back from stores…retailers will be like I don’t care if I’m charging more, people are still buying it to some degree. They’re just buying less. But all that money they’re spending is still going back into my store.” (Participant #8, Programme #7)*

Partnering with community organisations and community members has also been shown to be an effective method of letting stores know what their customers want. Examples of partnership included having an organised event where people from a community organisation would buy off the newly stocked healthy products.

Many of these suggested activities are rarely what funders and individuals envision when they think of HFRP. Thus, many participants advocated for finding and creating funding sources that allow for flexible spending. Some mentioned encouraging government and political backing as they can be the most reliable source of funding that one can have. Others suggest tapping into private funding that focusses more on small business development.

### Adolescent-specific recommendations

It was evident that participants largely had not specifically targeted adolescents in the HFRP that they were involved in. However, many suggested that activities that foster youth empowerment and sense of ownership are required for engaging adolescents. Convincing adolescents that they have the power to influence the stocking of foods in their local stores would better motivate healthier food purchasing behaviour than if they simply received instructions:

*“Kids love stuff like that, actually being involved in the process, and seeing how important it is for them to support their stores. Have them feel invested and empowered, kids love being treated like real adults. [Explain to them] this is how Bodega works, this is the issues with supply and demand. This is how you can support the Bodega. And what do you want to do? We had a contest, for the kids would come up with their own the three things that they would do to support the bodega, and then we would fund the one.” (Participant #10, Programme #6)*

Educational sessions related to nutrition can still play a role; however, they must be interactive, hands-on and can benefit from activities that capitalise on adolescents’ ‘rebellious’ tendencies. An example of such activities was described by one interviewee

*“The counter marketing is really helpful for that group. It’s generally the stereotype population that doesn’t like to be told what to do. If you tell them they’re being manipulated by marketers by the placement in the store, by the pricing, and explain why Beyonce is on the front of Pepsi to sell to them, I think they’re more likely to be a little more resistant to it.” (Participant #8, Programme #7),*

Finally, one should carefully think about targeting timing and food retailers that are most relevant for adolescents. In terms of timing, participants suggested focusing on before and after school, or even during lunchtime, as they have observed that these are the most popular times adolescents would purchase foods. In terms of food retailer types, most participants suggested focusing on corner stores near schools and potentially fast-food places.

## Discussion

This study is one of the few that have reported on facilitators and barriers to implementing HFRP. Overall, individuals who have been involved in implementing HFRP in NYC believed that while HFRP efforts have improved over the years, significant challenges remain. Relationship building with stores was pertinent to success, whereas securing reliable funding was one of the hardest barriers to overcome. Designing sustainable HFRP will require making programmes profitable, targeting shortcomings in distribution systems, and increasing demand.

While it requires substantial time and effort, building relationships with members of the community and store owners is essential to maintain credibility and support for the HFRP. This is consistent with findings from a collective case study of HFRP in supermarkets in several countries, including Australia, the Netherlands, and the UK^(55)^. During or before designing an HFRP, it is important to identify champions and/or store owners who are invested in providing healthy foods within the community if one wants to work in. These individuals are not rare; many store owners value their community’s health^(18,56)^ and can provide insight to their community’s needs and the struggles they face in stocking healthy foods^(57)^. Another avenue to relationship building is hiring locally based staff who share similar cultures with store owners, which has been shown to increase store buy-in^(56)^. A systematic review of USA-based food store interventions found that it is easier to build trust when HFRP staff share similar socio-cultural backgrounds with store owners^(18)^. Hiring staff from the community also promotes the local economy by creating workforce and elevating community capacity^(58)^.

Identifying champions and engaging stakeholders, such as store owners, are considered an integral part of the co-creation framework^(59,60)^. The framework describes a cross-sector collaboration approach that entails bringing together store owners, food distributors, government, consumers, as well as researchers and practitioners, to co-create HFRP that fulfil the shared interests of all involved^(59,60)^. This recently emerging framework has been suggested to create sustainable HFRP that improves the food environment more successfully compared with a ‘top-down’ approach, where external researchers independently design HFRP and then invite stores to participate in them^(61,62)^. Of particular note, the co-creation process has been found to increase trust between researchers, practitioners and community members^(60)^. Ultimately, the process leverages and prioritises the expertise of community members, which leads to an increased sense of ownership of the HFRP^(60,61)^.

HFRP often lack adequate support for store owners to improve their businesses. The tension between the desire to provide healthy foods and make a profit has been reported elsewhere^(57)^. Hence, an HFRP should be designed to generate profit for stores, which might require large capital investments to ensure that stores are equipped to stock healthy foods. A study examining fifty-seven small stores stressed the importance of technical and infrastructure to the stocking of healthy foods^(63)^. Funders should thus expand what they deem to be allowable as a budget item. Seeking alternative funding sources that focus on small business and community development, such as federal Community Economic Development (CED) programme^(64)^, may be another option. At present, many stores are provided equipment by companies that require exclusive stocking of their products, so supplying store owners with their own equipment encourages increased autonomy over the products they stock^(65,66)^.

This study highlighted the difficulty smaller stores face in stocking low-cost healthy foods within the context of the current distribution system. A study looking at corner stores’ network of suppliers in Baltimore, Maryland found that corner stores typically have fewer relationships with suppliers that can provide them with healthy foods^(67)^. To overcome the issue of small stores lacking connections and economy of scale, stores can collaborate with other retailers or become part of purchasing groups to generate purchasing power, as done in Detroit, Michigan, Minneapolis and Minnesota^(57)^. A study in Baltimore is ongoing in which retailers can utilise an app to purchase foods collectively with other retailers to reduce costs ^(68)^. A central location, such as food hubs, can be built where bulk purchases can be broken down for individual stores. Reducing the cost, time and effort for store owners to obtain healthier products could encourage them to self-stock such items^(18)^. New employment opportunities can be created to fulfil this need and merchandise healthy foods at stores. Placement and promotion of healthy foods in stores are a core part of increasing sales, especially in the face of promotions of less healthy products that are consistently maintained by salesman hired by large companies^(65,66)^.

Healthy food purchases depend on affordability and programmes that subsidise the cost of healthy foods can help. Expanding programmes such as Double Up Food Bucks, which matches the amount of Supplemental Nutrition Assistance Program (SNAP) dollar spent on fruits and vegetables, can fill this gap and indeed have been shown to increase fruits/vegetables purchase and intake^(69–71)^. These programmes can even be funded partly through taxing unhealthy competitive products, as in Seattle, Washington, where revenues were invested back to programmes such as Fresh Bucks and Farm to Table as well as early learning and child development programmes^(72)^. Philadelphia has a similar programme in which revenues from an SSB tax are fed back to pre-kindergarten programming^(73)^.

Interestingly, both Philadelphia’s and Seattle’s tax programmes focus on children, but HFRP rarely targets the youth, as was also evident in this study. However, adolescents regularly purchase foods/beverages independently^(31–34)^ and should be considered in HFRP work. Youth advocacy programmes were suggested to be a promising avenue to involve adolescents in developing HFRP. Others found that youth advocacy programmes promote health behaviour changes such as physical activity and tobacco cessation^(74,75)^. Involving youth in civic activities can also lead to policy changes as decision-makers may be more amenable to listening to youth’s requests^(76,77)^, and political will to intervene on the youth population is also typically greater than that for adults^(78)^.

This study has strengths and limitations. To our knowledge, this is one of the very few studies that have evaluated facilitators and barriers to HFRP success. These findings are shaped by a variety of stakeholders who have been involved in various parts of the programme’s process including designing, funding, implementing and evaluating HFRP. This allows for a more holistic view of potential strategies and issues that should be considered when planning HFRP. One limitation is that findings may not be generalisable to HFRP in other countries and other locations in the USA (e.g. rural areas), though they may be relevant to other urban cities with prominent corner store presence^(28,57)^. Additionally, recruitment of individuals who have worked with corner stores and supermarkets was prioritised, as most HFRP have targeted these food retailers. Thus, we cannot highlight the factors that may facilitate or bar success for programming in other types of food retailers, such as farmers’ markets, that also play a role in influencing healthy food access and availability^(71)^.

In summary, this study identified strategies that promote HFRP success and significant barriers that HFRP have faced. Extensive work is needed to ensure that stores can purchase affordable healthy foods from their distributor, which will translate to affordability for customers. A call for policies to support healthy food availability has been an ongoing effort^(28,58,79)^. Involving adolescents and highlighting the important role food stores have on adolescents’ health may help policymakers progress in this area. Future studies should work directly with adolescents and qualitatively explore the different factors influencing their purchasing behaviour and how HFRP can support them in purchasing healthy foods.
